# Environmental conditions modulate the effect of epigenetic factors controlling the response of *Arabidopsis thaliana* to *Plasmodiophora brassicae*

**DOI:** 10.3389/fpls.2024.1245545

**Published:** 2024-05-29

**Authors:** Mathilde Petitpas, Romane Lapous, Mathieu Le Duc, Christine Lariagon, Jocelyne Lemoine, Christophe Langrume, Maria J. Manzanares-Dauleux, Mélanie Jubault

**Affiliations:** IGEPP, Institut Agro Rennes-Angers – INRAE – Université de Rennes, Le Rheu, France

**Keywords:** DNA methylation, temperature rise, flood, drought, clubroot, plasticity

## Abstract

The resistance of *Arabidopsis thaliana* to clubroot, a major disease of Brassicaceae caused by the obligate protist *Plasmodiophora brassicae*, is controlled in part by epigenetic factors. The detection of some of these epigenetic quantitative trait loci (QTL^epi^) has been shown to depend on experimental conditions. The aim of the present study was to assess whether and how temperature and/or soil water availability influenced both the detection and the extent of the effect of response QTL^epi^. The epigenetic recombinant inbred line (epiRIL) population, derived from the cross between *ddm1-2* and Col-0 (partially resistant and susceptible to clubroot, respectively), was phenotyped for response to *P. brassicae* under four abiotic conditions including standard conditions, a 5°C temperature increase, drought, and flooding. The abiotic constraints tested had a significant impact on both the leaf growth of the epiRIL population and the outcome of the epiRIL–pathogen interaction. Linkage analysis led to the detection of a total of 31 QTL^epi^, 18 of which were specific to one abiotic condition and 13 common to at least two environments. EpiRIL showed significant plasticity under epigenetic control, which appeared to be specific to the traits evaluated and to the abiotic conditions. These results highlight that the environment can affect the epigenetic architecture of plant growth and immune responses and advance our understanding of the epigenetic factors underlying plasticity in response to climate change.

## 1 Introduction

Epigenetics studies the mechanisms involved in the modulation of gene expression without changes in DNA sequence (Waddington, 1959; Holliday, 1987). Transgenerational epigenetics focuses on the epigenetic marks that are not reset during mitotic or meiotic divisions and are therefore transmitted from one generation to the next (Heard and Martienssen, 2014; Quadrana and Colot, 2016). DNA methylation, i.e., the addition of a methyl group to the cytosine of the DNA sequence, is one of the most important epigenetic mechanisms involved in genome stability and modulation of gene expression (reviewed in Zhang et al., 2018). Methylation targets gene promoters, transposable elements, tandem, and interspersed repeats and occurs in three different contexts: symmetric CH and CHG and the rarest asymmetric CHH with H being either A, T, or C (Dowen et al., 2012; Deleris et al., 2016).

In the model species *Arabidopsis thaliana*, there is growing evidence that transgenerational epimutations, and in particular DNA methylation variations, are involved in the control of complex traits related to developmental processes (Johannes et al., 2009; Cortijo et al., 2014), metabolism (Aller et al., 2018), and response to abiotic (Kooke et al., 2015) and biotic stresses (Furci et al., 2019; Liégard et al., 2019). Indeed, as sessile organisms, plants are exposed to a wide range of pathogens that cause yield losses and threaten global food security (O’Brien et al., 2021). Climate change, especially fluctuations in temperature and water availability, affects both plant development and the response to pathogenic organisms (Barbetti et al., 2012; Bhadra et al., 2022). The extent to which environmental factors affect plants depends on the pathosystem, the intensity of the stress, and the duration of the stress application (for a review, see Desaint et al., 2021, and Velásquez et al., 2018). Furthermore and depending on the pathosystem, it has been shown that the application of an abiotic constraint can lead to improved resistance (Aoun et al., 2017) or increased susceptibility of a plant to a pathogen (Bidzinski et al., 2016). In addition, plants can adapt to environmental changes via phenotypic plasticity, which refers to the ability of one genotype to present different phenotypes under different environmental conditions (Bradshaw, 1965). Studying plastic responses to infection by pathogens would give us a better understanding of how to use (epi)genetic resistance in plant breeding to cope with climate change.

*Plasmodiophora brassicae* is a protist, soil-borne obligate pathogen and the causal agent of clubroot disease in the Brassicaceae family, which includes important economic crops (oilseed rape, cabbages, etc.) as well as *Arabidopsis* (Dixon, 2009). *P. brassicae* disrupts nutrient and water cycles, leading to yield loss and potential plant death (Dixon, 2009). After infection of the root hairs (primary infection) by primary zoospores released during germination of resting spores, secondary zoospores infect the cortical cells (secondary infection) of the root tissues and lead to gall formation through hyperplasia (cell division) and hypertrophy (cell enlargement) (Liu et al., 2020). The genetic architecture of the plant response to clubroot has been described in several Brassicaceae species (Manzanares-Dauleux et al., 2000; Rocherieux et al., 2004; Jubault et al., 2008; Lee et al., 2015), as well as the modulation of the effect of some genetic resistance factors by abiotic constraints such as nitrogen supply (Laperche et al., 2017; Aigu et al., 2018), water availability (Gravot et al., 2016), or soil pH (Gossen et al., 2012). Temperature was also found to significantly influence the outcome of *P. brassicae* infection (Sharma et al., 2011b, 2011a; Gossen et al., 2012). Interestingly, in *Arabidopsis*, natural and induced epigenetic quantitative trait loci (QTL^epi^) have been shown to be involved in the plant response to *P. brassicae* (Liégard et al., 2019; Gravot et al., 2024), with the detection of some of these QTL^epi^ being dependent on experimental conditions (Liégard et al., 2019).

In the present paper, we further investigated whether and how the epigenetic architecture of the plant response to clubroot depended on temperature and soil water content. To this end, the epigenetic recombinant inbred line (epiRIL) population (Johannes et al., 2009), derived from the cross between the Col-*ddm1-2* mutant (partially resistant to clubroot) and Col-0 (susceptible to clubroot) (Johannes et al., 2009; Liégard et al., 2019), was evaluated for clubroot resistance under different abiotic conditions: punctual and moderate temperature increase ( ± 5°C during night and day), water deficit, or excess water. Overall, our study confirmed that epigenetic variations were involved in controlling *A. thaliana* response to *P. brassicae* infection and highlighted that the environment can affect the epigenetic architecture of both plant growth and immune responses.

## 2 Materials and methods

### 2.1 Plant material

A total of 123 lines from the epiRIL population derived from the cross between the wild-type Colombia Col-0 and the EMS mutant Col-*ddm1-2* (Johannes et al., 2009) were provided by Versailles Arabidopsis Stock Center (http://publiclines.versailles.inrae.fr/). The parental line Col-*ddm1-2* did not germinate in all tests, which explains the lack of data for this genotype in some of our experiments. In order to validate the inoculation process and the pathotype of the *P. brassicae* isolate used, the following genotypes were used as part of the differential host set as characterised in Some et al. (1996): *Brassica napus* ssp. *oleifera* cv ‘Nevin’ (ECD6), *B. napus* ssp. *rapifera* cv ‘Wilhelmsburger’ (ECD10), *B. napus* ssp. *oleifera* (Brutor), *Brassica oleracea* ssp. *acephala* (C10, C7, and CB151), *Brassica rapa* ssp. *pekiniensis* cv ‘Granaat’ (ECD5), and *B. napus* cv ‘Mendel’.

### 2.2 Plasmodiophora brassicae

All pathological tests were carried out using the *P. brassicae* isolate *eH* (Fahling et al., 2003), which belongs to the pathotype P1 (Some et al., 1996). The inoculum was prepared at 10^7^ spores/mL as in Manzanares-Dauleux et al. (2000), and 1 mL of the inoculum was applied to the base of the seedlings 10 days post-sowing (stage 1.04; Boyes et al., 2001). The life cycle of *P. brassicae* in *A. thaliana* was completed in 21 days post-inoculation with the primary infection lasting 7 days and the secondary infection starting 7 days after infection until the end of the cycle (Liu et al., 2020).

### 2.3 Growth conditions and experimental set-up

The epiRIL response to clubroot was assessed under four environmental conditions in two growth chambers. The standard growth conditions used in our laboratory for pathological clubroot tests were used as the control environment (denoted STANDARD) with 22°C during the 16 h of light and 19°C during the 8 h of darkness and a volumetric water content (VWC; %) varying from 20% to 50% across the test. For the three other environmental conditions, plants were grown under the same conditions as in STANDARD, except from 7 to 14 days post-inoculation, which corresponds to the beginning of the secondary phase of infection (Liu et al., 2020), where the temperature or the soil water content was modified for 7 days. For the heat condition (denoted HEAT), a temperature increase of 5°C was applied (27°C during the light period and 24°C at night). For the moderate drought condition (denoted DROUGHT), watering was stopped 3 days before the start of the secondary cycle of *P. brassicae*, resulting in a decrease of the VWC from 30% to less than 10%. For the flooding condition (denoted FLOOD), plants were watered with 6 L of clear water for 7 days leading to water saturation and a VWC above 50%. These abiotic conditions were chosen so that the plant could perceive the stress applied while ensuring that both the plant and the pathogen could continue to grow. Finally, the 5°C temperature increase applied in this study corresponds to the increase predicted in the worst-case climate change scenario (IPCC, 2023).

For each environment, the epiRIL population as well as the parental lines were phenotyped in two biological replicates in a completely randomised block design (with two blocks per replicate, each block consisting of six plants per genotype). For each growth condition, 24 plants per epiRIL were thus phenotyped in response to clubroot infection. For each pathological test, seed germination was synchronised by placing seeds on wet blotting paper in Petri dishes for 2 days at 4°C in the dark. Seeds were sown individually in pots (7 × 7 × 8 cm) containing a soil mix of 54% peat, 40% sand, and 6% clay. The differential host set was repeated four times for each condition. For STANDARD, all four repetitions of the differential host set were placed under the conditions described for STANDARD. For HEAT, DROUGHT, and FLOOD, two repetitions were put in the STANDARD conditions, and the two others were placed in the conditions described for HEAT, DROUGHT, and FLOOD.

In order to distinguish the impact of the abiotic environment from that of the pathogen infection on the plant development, the epiRIL population was also grown under the four environmental conditions STANDARD, HEAT, DROUGHT, and FLOOD but without being inoculated by *P. brassicae* in two biological replicates in a completely randomised block design (with two blocks per replicate, each block consisting in three plants per genotype). For each growth condition, 12 plants per epiRIL were thus phenotyped without inoculation.

Ten Thermochron iButton Device (DS1922E/DS1921G, Maxim Integrated, San Jose, CA, USA) sensors per growth chamber were used to control the temperature, and six SenseCAP LoRaWAN (EU868Mhz, Seeed Studio, Mansfield, TX, USA) sensors were used for VWC assessment.

### 2.4 Phenotyping

The growth of plants infected or not infected by *P. brassicae* was assessed by measuring the length of the longest leaf of inoculated (Lfi) and non-inoculated plants (Lni). For each condition, measurements were taken 24 days post-sowing for one repetition and 27 days after sowing for the second repetition. These sampling times were considered as part of the design effect and were thus corrected using the linear model described in Section 2.5. dLf was denoted as the difference between Lfni and Lfi to assess the impact of the pathogen on plant growth. Plant response to *P. brassicae* infection was evaluated 3 weeks post-inoculation (31 days after sowing): plants were dug up and rinsed with clear water, and then the disease index (DI) described in Manzanares-Dauleux et al. (2000) was calculated, with DI = (n1 × 25 + n2 × 50 + n2 × 75 + n3 × 100)/N, where *ni* is the number of plants in the symptom class *i* and *N* the total number of plants assessed. Pictures of the plants were taken, enabling the acquisition of the gall area measure (variable GA) using FIJI (Schindelin et al., 2012). The DI is representative of the impact of clubroot on the root system, while the GA variable gives an indication of the size of the symptoms (gall size). A third disease-related trait (GALA), which expresses root disease symptoms relative to shoot development, was calculated as [GA/(Lfi^2^)] × 5,000, as defined in Gravot et al. (2011). Phenotypic plasticity, defined as the ability of plants to modulate their phenotype depending on their environment, was estimated by the difference between the value of each trait obtained under the STANDARD condition and that obtained in one of the other abiotic conditions tested (variable called deltaTrait). Thus, six deltaTraits were calculated: deltaLfni, deltaLfi, deltadLf, deltaDI, deltaGA, and deltaGALA. These variables were measured for the following comparisons: standard heat (StdHeat), standard drought (StdDrought), and standard flood (StdFlood).

### 2.5 Statistical analyses

A linear model was used to determine the effect of the epigenome and the effect of the experimental design for each trait assessed under the four environmental conditions using the following equation (**Supplementary Table 1**):


(1) $${\text{Y}}_{\text{i}\text{j}\text{k}}=\text{μ}+\text{E}{\text{G}}_{\text{i}}+{\text{R}}_{\text{j}}+{\text{B}}_{\text{k}(}{\text{R}}_{\text{j})}+{\text{e}}_{\text{i}\text{j}\text{k}}$$

where μ is the global mean, EG*i* is the epigenotype effect of the *i* line, *Rj* is the replicate effect, Bk(R*j*) is the block effect nested in the replicate effect, and e*ijk* is the residual fitted on a Gaussian distribution. The model was fitted using the function *lm* of the package *stats*, using R version 4.2.1 (R Core Team, 2022). For each trait, adjusted average values were computed from the results of Equation 1.

Analyses of variance were performed for each disease-related trait, and broad-sense heritability (*h* ^2^) was calculated as follows:


(2) $$h^{2}=\frac{Ge}{Ge+Gr}$$

where *Ge* is the estimated epigenetic variance and *Gr* is the estimated block × repetition effect. *Ge* and *Gr* were obtained from the linear model described in Equation 1.

Principal component analysis and clusterisation were achieved using all deltaTraits and were carried out using the R packages *FactoMineR* (Lê et al., 2008) and *Factoextra* (Kassambara and Mundt, 2020). All the comparisons between traits or conditions were achieved through the Kruskal–Wallis test unless stated otherwise.

### 2.6 QTL^epi^ detection

Methylation differences between the different lines were characterised by Colome-Tatche et al. (2012) using MeDIP-Chip and used to define 126 meiotically stable epigenetic markers. These epigenetic markers were then used to construct an epigenetic recombination map for subsequent linkage analysis studies (Colome-Tatche et al., 2012). QTL^epi^ were detected using the R package *qtl* (Broman et al., 2003) of the R 4.2.1 version (R Core Team, 2022). Single interval mapping (SIM) was first used to identify potential QTL^epi^ with the multiple imputation (imp) method using a step size of 2 cM and a window size of 10 cM. SIM thresholds were evaluated by doing 5,000 permutational tests, and significativity was fixed at α = 0.05. To improve the statistical power of the QTL^epi^ detection, multiple QTL mapping (MQM) (Arends et al., 2010) was achieved with the function *stepwise*. Logarithm of the odds (LOD) thresholds were calculated with 5,000 permutations using the *scantwo* function with a significance level of α = 0.05. The likelihood ratio of each trait was obtained using the *scantwo* function with 5,000 permutations for each trait and each abiotic condition (**Supplementary Table 1**). When QTL^epi^ were detected, the model was fitted with the *fitqtl* function to calculate the percentage of variation explained (R^2^) by each QTL^epi^. The *lodint* function was used to calculate the confidence interval of each QTL^epi^, and the function *effectplot* was used to estimate the epiallele effect. Finally, the *addint* function was used to assess putative interactions between detected QTL^epi^.

### 2.7 DNA sequence variation

To assess whether the QTL^epi^ detected were due only to epigenetic variations and not to genetic variations, the significance of the genetic variants present in the confidence interval of the QTL^epi^ was also tested. Whole-genome sequence data were available for the 123 epiRIL epigenomes (Gilly et al., 2014). GATK HaplotypeCaller 4.0 was used—in joint-genotyping mode—to identify single-nucleotide polymorphisms (SNPs) and indels (<100 bp) among 122 epiRILs. Raw variant calls were then filtered following GATK Best Practice suggestions and additional scripts. TE-Tracker (https://www.genoscope.cns.fr/externe/tetracker) was used to identify transposition events (TEs) exactly as previously described (Gilly et al., 2014; Liégard et al., 2019). In order to evaluate the impact of genetic variants on heritable variation in the response to clubroot, three QTL models were compared as described in Kooke et al. (2015): 1) using epigenetic markers at the QTL^epi^ peak, 2) using genetic DNA variants (SNP, indel, and TE insertion) located in the confidence interval instead of epigenetic markers, and 3) using both epigenetic and genetic variants.

## 3 Results

### 3.1 Effect of abiotic factors on the epigenetic architecture controlling foliar development

#### 3.1.1 Leaf growth of the epiRIL population was significantly affected by abiotic conditions

**Figure 1:**
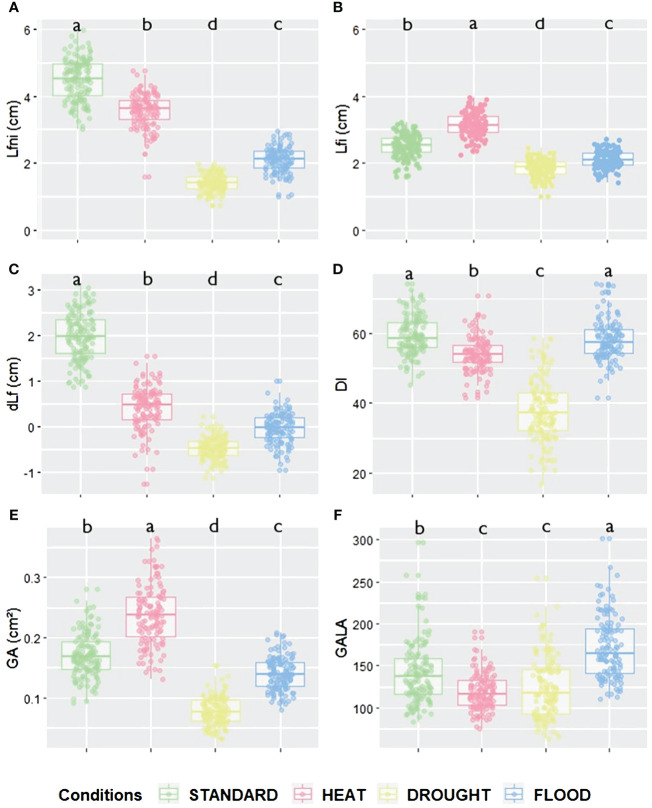
Leaf growth and disease-related trait values according to the different abiotic conditions for **(A)** Lfni, **(B)** Lfi, **(C)** dLf, **(D)** DI, **(E)** GA and **(F)** GALA variables. Green is for STANDARD, pink for HEAT, yellow for DROUGHT, and blue for FLOOD conditions. Significant differences for each trait between conditions are given by different letters obtained by a Kruskal–Wallis test, *p*-value<0.05.

In order to study the impact of the abiotic conditions HEAT, DROUGHT, and FLOOD on the epiRIL population, the length of the longest leaf of non-inoculated plants (Lfni) was measured as a proxy of leaf growth. Col-0 showed a significant reduction of Lfni by 45% in the FLOOD (Kruskal–Wallis test, *p* = 0.0087) and DROUGHT (*p* = 0.0012) conditions, while no significant effect was observed for the HEAT condition (**Supplementary Figure 1**). Data were not available for *ddm1-2*, as seeds did not germinate. The leaf growth of the epiRIL population was significantly affected by the HEAT, DROUGHT, and FLOOD conditions, resulting in a significant reduction compared to the STANDARD condition. HEAT had a small effect on leaf growth (20% reduction of Lfni, *p*< 0.001), while DROUGHT and FLOOD had a strong effect (68% and 53% reduction, respectively, *p*< 0.001) (**Figure 1A**, **Supplementary Tables 2**, **3**). In addition to the significant variability observed between conditions, leaf growth of the epiRIL population displayed variability within each abiotic condition with coefficients of variation ranging from 12.8% for STANDARD to 18.5% for DROUGHT.

#### 3.1.2 The epigenetic architecture controlling leaf growth was modified by abiotic conditions

**Figure 2:**
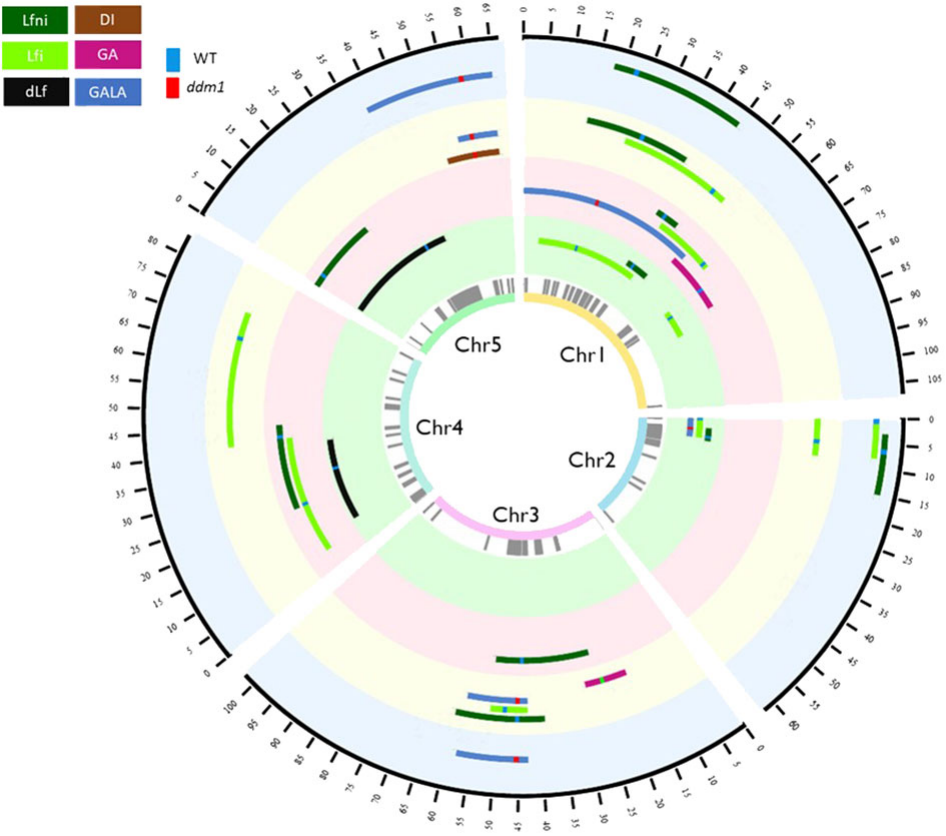
Circos representing the QTL^epi^ detected in the epiRIL population phenotyped for response to clubroot under four abiotic conditions. From the inner to the outer circle are indicated the chromosome number, differentially methylated regions used as epimarkers in grey, QTL^epi^ detected for the STANDARD condition, QTL^epi^ detected for the HEAT condition, QTL^epi^ detected for the DROUGHT condition, and QTL^epi^ detected for the FLOOD condition. The black outer circle represents the position in cM of the epigenetic map. Brown is associated with DI, pink with GA, blue with GALA, light green with Lfi, dark green with Lfni, and black with dLf variables. When methylation derived from the wild-type Col-0 is associated with higher values of the trait, the line at the marker at the peak of QTL^epi^ is green. When hypomethylation derived from the mutant *ddm1-2* is associated with higher values, the line at the marker at the peak of QTL^epi^ is red. QTL^epi^, epigenetic quantitative trait loci; epiRIL, epigenetic recombinant inbred line.

For the four environments tested, Lfni presented continuous distributions in the epiRIL population (**Supplementary Figure 1**), suggesting a poly-epigenetic control of this trait, with moderate-to-high broad-sense heritability (0.64 to 0.78, depending on the abiotic conditions; **Table 1** calculated according to Equation 2). In total, 13 moderate-effect QTL^epi^ (R^2^ ranging from 8.09% to 18.40%) controlling leaf growth were detected for all four conditions. Three QTL^epi^ were filtered out, as one genetic variant within the QTL^epi^ interval had a significantly larger effect than the epigenetic marker at the peak of the QTL^epi^ (**Supplementary Table 4**). Thus, 10 *bona fide* QTL^epi^ were conserved: two for STANDARD on chromosomes 1 and 2; four for HEAT on chromosomes 1, 3, 4, and 5; two for DROUGHT on chromosomes 1 and 3; and two for FLOOD on chromosomes 1 and 2 (**Figure 2**, **Table 2**). Several QTL^epi^ controlling Lfni overlapped and shared most of their confidence interval between conditions. Indeed, four QTL^epi^ overlapped on chromosome 1 for STANDARD, FLOOD, DROUGHT, and HEAT; two QTL^epi^ overlapped on chromosome 2 for STANDARD and FLOOD; and two overlapped on chromosome 3 for HEAT and DROUGHT. The phenotypic variability explained by these overlapping QTL^epi^ (R^2^) was consistent regardless of the abiotic conditions. Two epigenetic factors controlling Lfni were specific to the HEAT condition: one QTL^epi^ on chromosome 4 and one QTL^epi^ on chromosome 5.

**Table 1:** Broad-sense heritability (*h* ^2^) for leaf growth and disease-related traits in the epiRIL population under four abiotic conditions STANDARD, HEAT, DROUGHT, and FLOOD.

h²	STANDARD	HEAT	DROUGHT	FLOOD	Mean	Standard Deviation
**Lfni**	0.78	0.72	0.64	0.69	0.71	0.06
**Lfi**	0.71	0.68	0.66	0.76	0.70	0.04
**DI**	0.49	0.48	0.57	0.53	0.52	0.04
**GA**	0.66	0.55	0.58	0.62	0.60	0.05
**GALA**	0.67	0.63	0.47	0.52	0.57	0.09
**Mean**	0.66	0.61	0.58	0.62		
**Standard Deviation**	0.11	0.10	0.07	0.10		

Mean and Standard deviation for h^2^ are given within each condition and for each trait.

h² is the broad sense heritability calculated as followed:

$H^{2}=\;\frac{Ge}{Ge+Gr}$ with Ge is the estimated epigenetic variance and Gr is the estimated block x repetition effect.

Lfni, length of the longest leaf of uninoculated plants; Lfi, length of the longest leaf of inoculated plants; DI, disease index, GA, gall area and GALA: GA/Lfi^2^ index.

**Table 2:** Summary table of all the QTL^epi^ detected for STANDARD (green), HEAT (pink), DROUGHT (orange), and FLOOD (blue) conditions.

Conditions	Disease Related Traits	QTL^epi^ Names	MM	Chr	Pos (cM)	Lod	CI (cM)	Var (R², %)	Favourable allele	Type of QTL^epi^
**STANDARD**	Lfni	Lfni-Ch1-Standard	MM128	1	48	8.48	42-54	17.38	WT	Overlap
	Lfni	Lfni-Ch2-Standard	MM330	2	7.02	4.28	4.20-9.86	8.09	WT	Colocalise
	Lfi	Lfi-Ch1a-Standard	MM11	1	18.17	3.57	06-48	8.89	WT	Overlap
	Lfi	Lfi-Ch1b-Standard	MM160	1	70.09	4.242	68.36-78	10.68	WT	Specific
	Lfi	Lfi-Ch2-Standard	MM167	2	0.56	3.88	0-8.72	9.7	WT	Colocalise
	dLf	dLf-Ch5-Standard	MM724	5	34.97	3.99	2-43.98	11.85	WT	Specific
	dLf	dLf-Ch4-Standard	MM686	4	30	4.99	10-40	15.08	WT	Specific
	GALA	GALA-Ch2-Standard	MM171	2	5.33	3.95	0-8	13.74	*ddm1-2*	Specific
**HEAT**	Lfni	Lfni-Ch1-Heat	MM126	1	44.796	8.57	42-48	18.4	WT	Overlap
	Lfni	Lfni-Ch3-Heat	MM418	3	44.366	4.63	26.587-52	9.21	WT	Overlap
	Lfni	Lfni-Ch4-Heat	MM693	4	42.98	4.178	21.47-46	8.25	WT	Specific
	Lfni	Lfni-Ch5-Heat	MM707	5	4	4.64	0-14	9.25	WT	Specific
	Lfi	Lfi-Ch1-Heat	MM147	1	61.2	4.49	44.79-63.46	11.85	WT	Overlap
	Lfi	Lfi-Ch4-Heat	MM679	4	22	3.01	06-42	7.73	WT	Specific
	GA	GA-Ch1-Heat	MM159	1	68.36	3.95	54-74	13.43	WT	Specific
	GALA	GALA-Ch1-Heat	MM12	1	23.79	2.94	0-56	10.2	*ddm1-2*	Specific
**DROUGHT**	Lfni	Lfni-Ch1-Drought	MM25	1	29.04	5.21	16-40	15.84	WT	Specific
	Lfni	Lfni-Ch3-Drought	MM432	3	46.65	4.79	40-60	14.87	WT	Overlap
	Lfi	Lfi-Ch1-Drought	MM128	1	50	4.81	26.68-54	10.41	WT	Overlap
	Lfi	Lfi-Ch2-Drought	MM330	2	7.03	5.74	0-8.72	12.63	WT	Overlap
	Lfi	Lfi-Ch3-Drought	MM515	3	49.46	7.76	44.37-52.26	17.75	WT	Overlap
	Lfi	Lfi-Ch4-Drought	MM701	4	68.33	3	42-74	6.28	WT	Specific
	DI	DI-Ch5-Drought	MM863	5	59.93	2.52	52-65.93	8.13	*ddm1-2*	Specific
	GA	GA-Ch3-Drought	MM396	3	24	4.43	18-28	13.96	WT	Specific
	GALA	GALA-Ch3-Drought	MM432	3	46.65	4.27	44.37-58	12.54	*ddm1-2*	Colocalise
	GALA	GALA-Ch5-Drought	MM863	5	59.929	3.28	56.45-65.93	9.46	*ddm1-2*	Colocalise
**FLOOD**	Lfni	Lfni-Ch1-Flood	MM11	1	23.23	5.31	18-45.93	13.99	WT	Overlap
	Lfni	Lfni-Ch2-Flood	MM330	2	7.03	3.86	4.21-14.94	9.87	WT	Colocalise
	Lfi	Lfi-Ch2-Flood	MM167	2	0.557	3.58	0-8.72	14.57	WT	Colocalise
	GALA	GALA-Ch3-Flood	MM432	3	46.651	4.77	44.37-58	14.05	*ddm1-2*	Colocalise
	GALA	GALA-Ch5-Flood	MM863	5	59.93	3.11	39.49-65.93	8.9	*ddm1-2*	Colocalise

Information given: leaf growth and disease-related traits ordered by alphabetical order, the name of the QTL^epi^ (QTL^epi^ names), the molecular maker at the peak of the QTL^epi^ (MM), the chromosome (Chr), the confidence interval (CI), the position at the peak marker (Pos, cM), the phenotypic variance explained (Var, R^2^, %), the favourable allele being the allele associated with higher values, and the type of QTL^epi^. The QTL^epi^ were characterised as follows: the QTL^epi^ that are specific to one condition (specific), the QTL^epi^ that have a common confidence interval in at least two conditions (overlap), and the QTL^epi^ that have the same epimarker at their peak (colocalise).

QTL^epi^, epigenetic quantitative trait loci.

### 3.2 The abiotic conditions significantly modulated the interaction between *A. thaliana* and *P. brassicae*

To fully assess the plant response to clubroot, several traits were measured. As previously conducted on non-inoculated plants, the leaf growth of the inoculated plants was estimated by measuring the length of the longest leaf (Lfi). The effects of *P. brassicae* on plant leaf growth were assessed by calculating the variable dLf. GA and DI assessing the gall symptom size and the severity of the disease, respectively, allowed the evaluation of clubroot symptoms and root system functionality. As epiRIL showed different leaf growth under STANDARD conditions, and moreover the same epiRIL can show differences between the abiotic conditions, the GALA index, which considers leaf growth (Lfi) as a function of symptom size (GA), was calculated to compare symptoms between epiRIL in the same condition and between epiRIL in the different abiotic conditions. For each experiment, proper inoculation and the pathotype P1 of the *eH* isolate used were confirmed using the differential host set under the condition STANDARD. The symptoms observed on the differential hosts under HEAT and FLOOD conditions were similar to those observed under the STANDARD condition. Under the DROUGHT condition, the differential host set showed a significant reduction in disease symptoms for all susceptible genotypes, with, for example, a reduction of 32% in disease severity for the susceptible control accession ECD5 (Wilcoxon test, *p* = 0.0015) (**Supplementary Table 5**).

The response of Col-0 and *ddm1-2* to clubroot was differently influenced by the abiotic factors. While neither FLOOD nor HEAT had a significant impact on the response of Col-0, the DROUGHT condition led to a significant reduction of leaf growth (32% decrease, *p* = 0.029), symptom severity (33% decrease; Kruskal–Wallis, *p* = 0.042), and root biomass (56% decrease, *p* = 0.029) compared to the STANDARD condition. For *ddm1-2*, leaf growth was positively affected by HEAT (increase for Lfi by 53%, *p* = 0.029), negatively affected by DROUGHT (Lfi reduced by 26%, *p* = 0.029), and not impacted by FLOOD; both disease severity and gall size were negatively affected by DROUGHT (reduction of DI by 65%, *p* = 0.042, and GA by 62%, *p* = 0.029). Only the HEAT condition resulted in a significant reduction in symptom size standardised by leaf growth, as the GALA index was reduced by 46% (*p* = 0.029) (**Supplementary Figure 1**).

The response of the epiRIL population to clubroot was also significantly impacted by abiotic factors. Indeed, compared to the STANDARD condition, the HEAT condition resulted in a significant increase in leaf growth (25% increase in Lfi, *p*< 0.001) and gall size (39% increase in GA, *p*< 0.001) and a significant decrease in disease severity and symptom size normalised by leaf growth (DI reduced by 9%, *p*< 0.001, and GALA reduced by 15%, *p*< 0.001) (**Figure 1**; **Supplementary Tables 2**, **3**). The DROUGHT and FLOOD conditions resulted in a significant decrease in leaf growth (26% and 16% reduction in Lfi, respectively, both *p*< 0.001). DROUGHT led to a significant reduction in disease severity (DI reduced by 36%, *p*< 0.001), gall size (GA reduced by 54%, *p*< 0.001), and symptoms size normalised by leaf growth (GALA reduced by 11%, *p*< 0.001). In the FLOOD condition, galls were significantly smaller (GA reduced by 18%, *p*< 0.001), but disease severity was not affected, and symptom size standardised by leaf growth increased significantly (GALA increased by 22%, *p*< 0.001). The disease index variable (DI) did not display much variability across abiotic conditions. In the STANDARD condition, *P. brassicae* significantly reduced leaf growth, as dLf was equal to an average of 1.98 ± 0.52 cm. For the other conditions, however, the impact of the pathogen on leaf growth was almost negligible, as dLf was equal to an average of 0.42 ± 0.45 cm, −0.47 ± 0.24 cm, and −0.03 ± 0.32 cm for HEAT, DROUGHT, and FLOOD, respectively (**Figure 1**, **Supplementary Tables 2**, **3**).

Regardless of the conditions, the positive correlation between leaf growth and gall size was maintained (Pearson’s r = 0.6), highlighting that gall development was not independent of leaf growth and that the Lfi and GA traits may be under the control of common epigenetic factors (**Supplementary Figure 2**). The existence of a positive or negative correlation between variables may depend on abiotic conditions, as illustrated by the correlation between disease index and gall size, which was positive under the DROUGHT conditions (Pearson’s r = 0.39) and negative under the STANDARD conditions (Pearson’s r = −0.29).

### 3.3 The epigenetic architecture controlling the response of epiRIL to clubroot is modified by abiotic constraints

For all abiotic conditions, the distributions of disease-related variables were continuous, suggesting a poly-epigenetic control of all traits (**Supplementary Figure 1**). Broad-sense heritability values ranged from moderate to high (**Table 1**), indicating that most part of the phenotypic variation observed was explained by the epigenotype. Twenty-three QTL^epi^ were detected for all abiotic conditions and all traits, but two of them were eliminated because the closest genetic variants had a significantly larger effect than the epigenetic markers at the QTL^epi^ peak (**Supplementary Table 4**). Twenty-one moderate-effect QTL^epi^ were then considered *bona fide* epigenetic QTL controlling plant response to clubroot in all conditions (**Figure 2**, **Supplementary Table 4**). The number of QTL^epi^ detected for each condition was different: six QTL^epi^ were detected for STANDARD, four for HEAT, eight for DROUGHT, and three for FLOOD. Very few QTL^epi^ were detected for the variables dLf (two QTL^epi^ detected under STANDARD condition) and DI (one QTL^epi^ detected in the DROUGHT condition), which is consistent with the low phenotypic variability observed for these two traits in the epiRIL population. Regardless of the abiotic conditions, the epiRIL carrying the hypomethylated allele derived from *ddm1-2* showed greater symptoms standardised by leaf growth (higher GALA) and greater disease severity (higher DI) while displaying smaller rosettes (smaller Lfi). With the exception of two QTL^epi^ (Lfi-Ch1b-STANDARD and Lfi-Ch4-DROUGHT), all Lfi QTL^epi^ overlapped (i.e., they shared most of their confidence interval) with QTL^epi^ detected for Lfni. Further details on the QTL^epi^ localisations and confidence intervals are given in **Table 2**.

Eight QTL^epi^ colocalised (we considered that two QTLs colocalised when they had the same epigenetic marker at the peak of the QTL) and 10 QTL^epi^ overlapped between at least two abiotic conditions (**Table 2**). Some of the QTL^epi^ detected under the STANDARD condition were also detected in other conditions, in particular for Lfi, in two genomic regions located on chromosomes 1 and 2. Other QTL^epi^, not detected in STANDARD, were found in at least two abiotic conditions, in particular for GALA, for which four QTL^epi^ colocalised between DROUGHT and FLOOD on chromosomes 3 and 5. Finally, 13 QTL^epi^ were specific to one abiotic condition. For Lfi, one QTL^epi^ on chromosome 1 was specific to STANDARD, two on chromosomes 3 and 4 were specific to DROUGHT, and one on chromosome 4 was specific to HEAT. For GA, two QTL^epi^ were specific to HEAT and DROUGHT on chromosomes 1 and 3. For GALA, two QTL^epi^ were specific to HEAT and STANDARD on chromosomes 1 and 2. For DI, one QTL^epi^ on chromosome 5 was specific to DROUGHT.

### 3.4 Epigenetic factors involved in the control of phenotypic plasticity

We examined in more detail the phenotypic plasticity observed within the epiRIL population between STANDARD and the other three abiotic conditions, that is to say, the ability of an epigenotype to modulate its phenotype as a function of the environment. Plasticity was estimated by the difference between the value of each trait obtained under the STANDARD condition and that obtained under another abiotic condition. For each trait, the correlation between conditions was assessed. For the GALA index, a correlation of 1 was observed between the DROUGHT and FLOOD conditions. For the other traits, the correlation coefficients between the conditions ranged from 0.282 to 0.689 (**Supplementary Figure 3**).

#### 3.4.1 EpiRIL responded differently to clubroot depending on abiotic conditions

**Figure 3:**
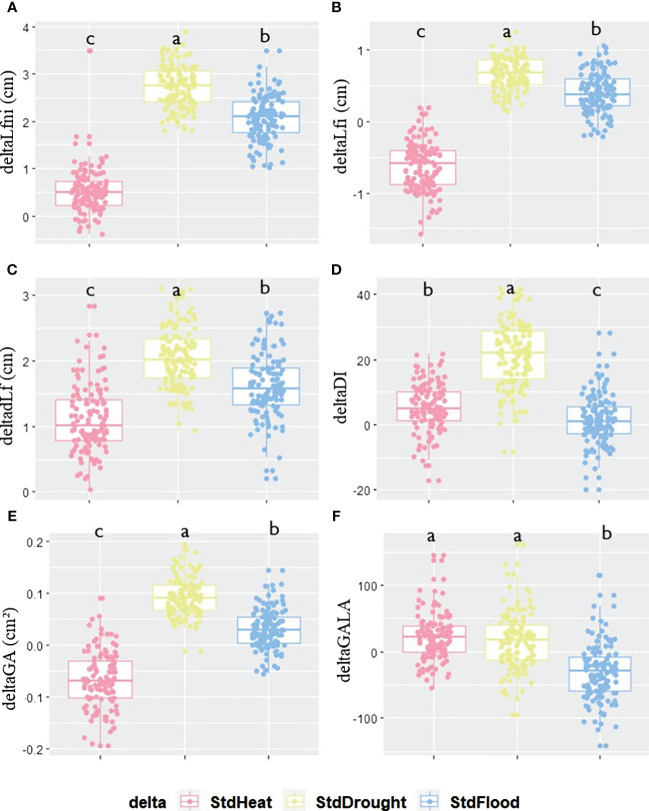
Phenotypic plasticity calculated as the difference between the value of each trait evaluated under STANDARD conditions and that assessed under one of the three other conditions: HEAT, DROUGHT, or FLOOD. Differences between STANDARD and HEAT (pink), STANDARD and DROUGHT (yellow), or STANDARD and FLOOD (blue) for each trait for **(A)** deltaLfni, **(B)** deltaLfi, **(C)** deltadLf, **(D)** deltaDI, **(E)** deltaGA, and **(F)** deltaGALA variables. Significant differences for each deltaTrait between conditions are given by different letters obtained by a Kruskal–Wallis test, *p*-value<0.05.

The two parental lines did not show similar phenotypic plasticity, neither for phenotypic traits nor between abiotic conditions (**Supplementary Figure 4**). With respect to all deltaTraits, Col-0 and *ddm1-2* showed similar plasticity in HEAT conditions (mean of absolute delta = 22 ± 16.89 and 31 ± 18.88 for StdHeat, respectively). Under the DROUGHT condition, both *ddm1-2* (mean delta = 39 ± 23.84 for StdDrought) and Col-0 (mean delta = 59 ± 43.33 for StdDrought) showed great phenotypic plasticity except for deltaGALA for which values ranges from 10.8 to 14.4. For FLOOD, Col-0 displayed greater phenotypic plasticity (mean absolute delta = 41 ± 30.74 for StdFlood) than *ddm1-2* (mean delta = 18 ± 13.33 for StdFlood). The influence of abiotic factors on the response of the epiRIL population to clubroot was not the same under all conditions (**Figure 3**; **Supplementary Figure 5**, **Supplementary Table 6**). For deltaLfni, deltaLfi, and deltadLf, low phenotypic plasticity was observed when comparing STANDARD and HEAT, whereas high plasticity was found under StdDrought and StdFlood (**Figures 3A–C**). For deltaDI and deltaGA, phenotypic plasticity was high for StdDrought and low for StdHeat and StdFlood (**Figures 3D, E**). For deltaGALA, plasticity was low for StdHeat and StdDrought and high for StdFlood (**Figure 3F**).

**Figure 4:**
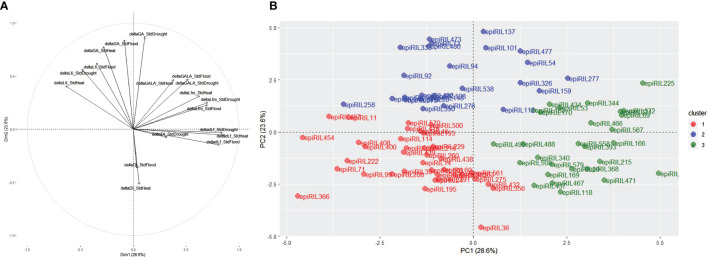
**(A)** Principal component analysis (PCA) displaying distributions of deltaTraits and **(B)** *k*-means clustering based on the PCA carried out for all deltaTraits for all conditions for the epiRIL with a cos^2^ higher than 0.4. epiRIL, epigenetic recombinant inbred line.

In order to find lines that were phenotypically more stable (deltaTraits close to 0) or more plastic (high absolute values of deltaTraits) in their response to *P. brassicae* regardless of abiotic conditions, principal component analysis (PCA) and *K-means* clusterisation were achieved using deltaTraits calculated between STANDARD and HEAT, DROUGHT, and FLOOD (**Figures 4A, B**). To facilitate the analysis and retain epiRIL with extreme phenotypes, the best quality epiRIL on the PCA was retained (square cosine >0.4). For each abiotic trait, the correlation between SdtHeat, StdDrought, and StdFlood was observed (**Figure 4A**). The first principal component was explained by deltaLfni and deltadLf, while the second principal component was mainly explained by deltaLfi and deltaGA. Ninety-one lines were kept and spread within three clusters (**Figure 4B**). EpiRIL in the first cluster displayed less plasticity regardless of the trait than the lines in the two other clusters, while lines in the second cluster displayed higher plasticity for GA and Lfi, and lines in the third cluster showed higher plasticity for dLf, Lfni, and GALA.

For each deltaTrait, the observed phenotypic plasticity varied moderately between abiotic conditions according to the deltaTraits. For deltaLfni, for example, lines belonging to the first cluster were less plastic for all abiotic conditions. For deltaLfi, lines belonging to the second cluster were less plastic under StdHeat, whereas for StdFlood and SdtDrought, the less plastic lines belonged to the third cluster (**Supplementary Figure 6**).

#### 3.4.2 Phenotypic plasticity in the epiRIL clubroot response is partly controlled by epigenetic factors

The distributions of all deltaTraits were continuous in the epiRIL population (**Supplementary Figure 4**). Ten moderate-effect QTL^epi^ were found to control phenotypic plasticity (**Table 3**), and all were *bona fide* QTL^epi^ (**Supplementary Table 7**). No QTL^epi^ were detected for StdFlood. One QTL^epi^ was detected for DI plasticity between STANDARD and HEAT and one for Lfi and Lfni plasticity between STANDARD and DROUGHT; three QTL^epi^ were detected for GALA plasticity between STANDARD and HEAT, and four QTL^epi^ were detected for GALA plasticity between STANDARD and DROUGHT. An epistatic interaction was found between two QTL^epi^ controlling GALA plasticity under HEAT on chromosomes 1 and 5 (**Table 3**). EpiRIL carrying a Col-0 derived hypermethylation allele showed lower deltaGALA and deltaDI for StdHeat while displaying higher deltaLfni, deltaLfi, and deltaGALA for three out of four QTL^epi^ for StdDrought (**Supplementary Figure 7**). For StdDrought, similar epigenetic control was observed for Lfi, Lfni, deltaLfi, and deltaLfni. Indeed, all QTL^epi^ detected for deltaLfi and deltaLfni overlapped with QTL^epi^ detected previously for Lfni and Lfi. For deltaGALA, four out of seven QTL^epi^ detected overlapped with QTL^epi^ detected for GALA. No QTL^epi^ detected for deltaDI overlapped with QTL^epi^ detected for DI (**Supplementary Figure 8**). Explained phenotypic variability by the detected QTL^epi^ was similar for Lfni/deltaLfi and Lfi/deltaLfi (R^2^ Lfi = 16.8 ± 0.8; R^2^ Lfni = 9.9 ± 0.8; R^2^ deltaLfi = 9.55; R^2^ deltaLfni = 17.12) as well as for GALA (R^2 =^ 18.84 ± 2.5) and deltaGALA (15.63 ± 8.1).

**Table 3:** Summary table of all the QTL^epi^ detected for deltaDI_StdHeat, deltaGALA_StdHeat, deltaLfi_Drought, deltaLfni_Drought, and deltaGALA_StdDrought variables.

Conditions	Disease Related Traits	QTLepi Names	MM	Chr	Pos (cM)	Lod	CI (cM)	Var (R², %)	Favourable Allele	PairWise Interaction
**StdHeat**	deltaDI_StdHeat	deltaDI_StdHeat_Ch1	MM2	1	1.73	2.7	0-24	9.39	*ddm1-2*	NA
	deltaGALA_StdHeat	deltaGALA_StdHeat_Ch1	MM160	1	70	7.55	68.35-76	22.44	*ddm1-2*	deltaGALA_StdHeat_Ch5
	deltaGALA_StdHeat	deltaGALA_StdHeat_Ch2	MM171	2	5.34	2.62	0-12	7.09	*ddm1-2*	NA
	deltaGALA_StdHeat	deltaGALA_StdHeat_Ch5	MM718	5	31	5.73	12-32.73	5.73	*ddm1-2*	deltaGALA_StdHeat_Ch1
**StdDrought**	deltaLfi_StdDrought	deltaLfi_StdDrought_Ch1	MM10	1	18.18	2.75	6-84	9.55	WT	NA
	deltaLfni_StdDrought	deltaLfni_StdDrought_Ch1	MM128	1	45.93	5.14	42-54	17.12	WT	NA
	deltaGALA_StdDrought	deltaGALA_StdDrought_Ch2a	MM373	2	10.97	7.97	10.41-12	23.43	*ddm1-2*	NA
	deltaGALA_StdDrought	deltaGALA_StdDrought_Ch2b	MM374	2	12	7.43	10.97-13.25	21.59	WT	NA
	deltaGALA_StdDrought	deltaGALA_SdtDrought_Ch3	MM432	3	46.65	3.83	46-48.32	10.4	WT	NA
	deltaGALA_StdDrought	deltaGALA_StdDrought_Ch4	MM679	4	22.02	3.42	0-38	9.19	WT	NA

Information given: disease-related traits ordered by alphabetical order, the name of the QTL^epi^ (QTL^epi^), the molecular maker at the peak of the QTL^epi^ (MM), the chromosome (Chr), the confidence interval (CI), the position at the peak marker (Pos, cM), the phenotypic variance explained (Var, R^2^, %), the favourable allele being the allele associated with higher values, and the type of QTL^epi^ and the names of QTL^epi^ in interaction.

QTL^epi^, epigenetic quantitative trait loci.

## 4 Discussion

In the present study, we investigated the impact of three abiotic conditions on the response of *A. thaliana* against the protist *P. brassicae* and to what extent the epigenetic control of *Arabidopsis* response to clubroot was impacted by these abiotic conditions. To that aim, the epiRIL population of Johannes et al. (2009) was phenotyped under three abiotic conditions never before studied for this population: a punctual and moderate rise of temperature, a drought, or a flood. These abiotic conditions led to a significant modification in *Arabidopsis* leaf growth, its response to clubroot, and the epigenetic factors controlling these traits. This work also enabled the identification of QTL^epi^ underlying the plastic response of *A. thaliana* to clubroot, which depended on the phenotypic traits assessed and the abiotic conditions. This study allowed us to extend our knowledge of the impact of abiotic conditions on the epigenetic factors controlling both plant responses to biotic and abiotic stresses and phenotypic plasticity.

### 4.1 A small number of regions control the response to clubroot in the epiRIL population whatever the growing conditions

Five to 10 QTL^epi^ per condition and one to 10 QTL^epi^ per trait were identified as involved in *A. thaliana* response to clubroot under the four different abiotic conditions. This small number of QTL^epi^ can be explained by the lack of phenotypic variability observed for disease-related traits. Indeed, the 123 epiRIL studied were selected by Colome-Tatche et al. (2012) for their contrasted morphological traits and not for their response to pathogens. The small size of the epiRIL population (123 lines), the low number of epigenetic markers available (126 markers), and the bias in the segregation of the Col-0 (73%) and *ddm1-2* (27%) epialleles may also have led to a lack of power in the linkage analyses.

Furthermore, the length of the longest leaf in each rosette was the only proxy used to estimate leaf growth; other criteria, such as leaf mass, leaf area, or even photosynthetic ratio per leaf area, were not taken into account. According to Weraduwage et al. (2015), the relationship between leaf area growth and plant growth is not necessarily linear, as it depends on carbon distribution in the plant tissues. This suggested that the use of the Lfni variable cannot reflect alone the complexity of leaf growth. For example, Kooke et al. (2015) evaluated the impact of salt stress on *Arabidopsis* development using several variables such as relative growth rate (RGR), leaf area at 20 days (LA20), and total plant height (TPH). Despite these limitations, measuring the length of the longest leaf enabled us to detect differences between conditions. The reduction in leaf size between the STANDARD and the other conditions also allowed us to validate that the plants perceived the constraints applied.

The small number of QTL^epi^ detected within our study is coherent with the low number of QTL^epi^ detected by Kooke et al. (2015) using the same epiRIL population tested under saline conditions. For example, LA20 was controlled by five and four QTL^epi^ under control and saline conditions, respectively. Furthermore, the epigenetic markers (MM11 and MM160) at the peak of two of the five QTL^epi^ detected in our study controlling leaf growth (Lfni and Lfi) were also those detected at the peak of the QTL^epi^ in the study of Kooke et al. (2015). This may suggest that plant development was controlled by common epigenetic factors regardless of abiotic conditions. The lines carrying the hypomethylated allele were smaller than the lines carrying the hypermethylated allele for most leaf growth QTL^epi^, which is consistent with the growth abnormalities observed for the homozygous *ddm1-2* mutant (Kakutani et al., 1996).

### 4.2 Abiotic constraints significantly impact leaf growth and the outcome of the epiRIL–pathogen interaction

The abiotic constraints applied were chosen according to the characteristics and needs of both *A. thaliana* and the pathogen *P. brassicae*. Only a moderate drought was thus applied, as the zoospores of *P. brassicae* require high soil moisture to move and properly infect the plants (Aist and Williams, 1971; Iwama et al., 1994). Although the plant displayed reduced leaf growth, no wilting was observed, confirming that the drought applied is a moderate constraint for the plant (Bac-Molenaar et al., 2016). A previous study also demonstrated that soil water content, and more particularly flooding during 14 days, had an impact on both the outcome of the infection and the genetic factors involved in plant response to clubroot (Gravot et al., 2016). We applied a FLOOD constraint of only 7 days in contrast to Gravot et al. (2016), which led to a reduction in leaf growth and an enhanced production of anthocyanins, suggesting a plant response to oxidative stress caused by the water excess (Nakabayashi et al., 2014; Savchenko et al., 2019). Finally, the increase of 5°C for 7 days, which also led to a reduction of leaf growth, corresponds to a warm ambient temperature as described in Sriden and Charoensawan (2022) and is in accordance with the worst-case climate change scenario, which estimates for 2,100 an increase of more than 4°C (IPCC, 2023).

Comparison of the results obtained with literature data is complicated due to the diversity of conditions under which abiotic stresses were exerted in terms of duration, intensity, and time of application as well as the sequential or concomitant application of these stresses. In our study, increasing temperature to 27°C during the day and 24°C at night, for 7 days, led to a significant decrease in leaf growth. A temperature of 28°C had a significant negative effect on the total biomass of *Arabidopsis* (Jin et al., 2011) with a bigger hypocotyl and reduced developmental time (Hwang et al., 2017; Ibañez et al., 2017). Decreased plant growth, flowering time, and yield are commonly observed for several crops, such as wheat or oilseed rape at 29.5°C (Morrison and Stewart, 2002; Mondal et al., 2013). The DROUGHT and FLOOD conditions also led to a significant decrease in leaf growth in our study. Drought caused a reduction in leaf size in *Medicago sativa* (Quan et al., 2016), *B. napus* (Batool et al., 2023), and *A. thaliana* (Tisné et al., 2013; Bac-Molenaar et al., 2016, p.) as flooding in *A. thaliana* (Pigliucci, 2002). Concerning the epigenetic architecture controlling response to HEAT, DROUGHT, and FLOOD, we have detected a maximum of 10 moderate-effect QTL^epi^ (R^2^ between 8% and 20%). A small number of moderate-effect QTLs have also been found to control plant response to water deficit (Tisné et al., 2013), as well as major genes in the control of plant tolerance to flooding (Akman et al., 2017) or drought (MarChadier et al., 2019).

Depending on the sources of resistance and the isolates studied, clubroot resistance in Brassicaceae is controlled by a few major resistance loci (Manzanares-Dauleux et al., 2000; Laperche et al., 2017; Aigu et al., 2018) or a small number of QTL/QTL^epi^ with moderate effects (Rocherieux et al., 2004; Liégard et al., 2019), as in the present study. The epiRIL population was phenotyped for response against *Hyaloperonospora arabidopsidis*, a downy mildew pathogen of *Arabidopsis* (Furci et al., 2019), but no QTL^epi^ was detected in common between our two studies, suggesting that the epigenetic factors involved in plant response to biotic stresses are pathogen-specific. It would be interesting to study the epigenetic determinism of the epiRIL response to other isolates of *P. brassicae* and other pathogens in order to determine the specificity of the detected epigenetic factors and their interest in the control of immunity in *Arabidopsis*.

For the first time, the impact of three abiotic constraints was studied on plant clubroot response and the underlying epigenetic factors controlling these traits using the epiRIL population. The importance of taking environmental factors into account for studying plant resistance to biotic attacks was highlighted in the 1960s (Walker, 1965), but to date, no trend could be drawn, as the impact of abiotic factors on plant response to pathogens is complex. Indeed, the outcome of the plant–pathogen interaction depends on the plant, the pathogen, as well as the environment, and the duration and the way in which the abiotic constraints are applied (Velásquez et al., 2018; Desaint et al., 2021; Zandalinas and Mittler, 2022). Under combined abiotic constraints, the plant physiological response observed is different than the response observed under one stress (Suzuki et al., 2014). Assessing the impact of combined biotic and abiotic stress is all the more complex as the pathogen can also be impacted by the abiotic stress (Pandey et al., 2015). For instance, the temperature of 27°C that we used in the HEAT condition is above the optimum temperature for the growth of *P. brassicae*, which is 25°C (Sharma et al., 2011a); similarly, the DROUGHT conditions may have impaired *P. brassicae* life cycle (infection of the cortex cells by the secondary spores), as both the epiRIL population and the susceptible *Brassica* ECD5 showed lower susceptibility. This lower susceptibility to clubroot may indeed be explained by a lower infection of plants by *P. brassicae* due to the poor survival and/or motility of zoospores in a dry environment (Aist and Williams, 1971; Iwama et al., 1994) but also by the obligatory biotrophic nature of *P. brassicae*, whose development therefore depends on that of its host plant (Rolfe et al., 2016; Schwelm et al., 2016). Depending on the abiotic constraints, their application, and the pathosystem, abiotic constraints can lead to a decrease or an increase in symptoms. Under drought, a decrease in tomato grey mould infection (Achuo et al., 2006) and an increase in the susceptibility of common bean to charcoal rot disease (Mayek-Pérez et al., 2002) were observed. Taken together, these results strongly highlight the importance and complexity of the impact of abiotic factors on plant–pathogen interactions since abiotic factors can impair the development of both plants and pathogens and their interaction.

### 4.3 Response to clubroot depends on both QTL^epi^ stable regardless of the abiotic conditions and QTL^epi^ specific to each abiotic condition

Epigenetic factors controlling response to clubroot were specific to one or several abiotic conditions. We were able to detect 18 QTL^epi^ common to at least two abiotic conditions and 13 QTL^epi^ specific to the abiotic condition applied, suggesting that the epigenetic architecture controlling response to clubroot includes both QTL^epi^ stable regardless of the abiotic conditions and QTL^epi^ specific to each abiotic condition. We cannot compare these results to the literature because, to our knowledge, no other work has been conducted on the impact of abiotic factors on the epigenetic architecture controlling plant response to biotic stresses. However, in several works dealing with genetic control of resistance, the same genetic factors of resistance were detected under different abiotic conditions. In a previous study, we showed that two resistance QTL to clubroot were systematically detected regardless of the nitrogen supply but had a different effect depending on the nutritional constraint (Laperche et al., 2017). Galiano-Carneiro et al. (2021) reported the stability of genetic factors involved in corn resistance to leaf blight across multi-environmental conditions. On the contrary, in *A. thaliana*, new QTLs resistant to *Ralstonia solanacearum* were detected under conditions of increased temperature (Aoun et al., 2017). All QTL^epi^ detected in all conditions in our study displayed similar moderate effects. However, we cannot completely exclude the possibility of modulation of the QTL^epi^ effect by abiotic constraints since the lack of detection power in the epiRIL population could prevent the detection of weak-effect QTL^epi^. Thus, QTL^epi^ qualified as specific must therefore be taken with caution, and additional work is necessary to evaluate and confirm their specificity. Only single abiotic constraints were assessed in this work, but studying the combined effect of several abiotic stresses or with a different application time on the resistance to a pathogen could allow us to better understand the plant response to real and fluctuating environmental constraints.

The majority of QTL^epi^ were located within the pericentromeric regions of *Arabidopsis* chromosomes, most likely due to the involvement of *DDM1* in maintaining transposable element methylation (Cortijo et al., 2014) in the pericentromeric regions. However, three QTL^epi^ (GALA-Ch5-DROUGH, GALA-Ch5-FLOOD, and DI-Chr5-DROUGHT) were also identified outside these regions. These QTL^epi^ overlapped with *Pb-At5.2*, a QTL previously detected by Jubault et al. (2008) using a RIL population from the cross Col-0 × Bur-0 and whose effect was modulated by the water irrigation regime (Gravot et al., 2016). *Pb-At5.2* QTL fine-mapping led to the identification of two causal NLR genes *AT5G47260* and *AT5G47280* controlled by a natural epimutation (Gravot et al., 2024). However, because these *AT5G47260*/*AT5G47280* genes are 500 kbp distant from the MM863 epimarker at the peak of the three QTL^epi^ and a large number of genes in the QTL^epi^ confidence interval (more than 900 genes), we could not conclude whether or not it is the same causal gene(s). The QTL^epi^ at the end of chromosome 5 also overlapped with a QTL^epi^ at the epimarker 859 controlling short-chain glucosinolate levels (Aller et al., 2018), even if no candidate gene involved in glucosinolate biosynthesis pathway was found in the QTL^epi^ detected by Aller et al. (2018). Further work is therefore needed to identify the causal genes underlying these QTL^epi^.

### 4.4 Clubroot response plasticity of the epiRIL population

Phenotypic plasticity was assessed as the ability of individual epiRIL to modulate their phenotype according to environmental conditions (Bradshaw, 1965). For each genotype, plasticity was found to be dependent on both the abiotic conditions applied and the traits assessed. Phenotypic plasticity was brought depending on the traits observed by both hyper- and hypomethylated alleles, while previous work on saline stress associated more plasticity with the lines carrying the hypermethylated allele at the epigenetic marker of the QTL^epi^ (Kooke et al., 2015). While the QTL^epi^ controlling developmental plasticity overlapped with the QTL^epi^ detected for the developmental trait (leaf length), the QTL^epi^ controlling the disease-related trait plasticity were different from those controlling the associated phenotypic trait, suggesting that different factors are involved in the control of plasticity and their related traits. In *A. thaliana*, in a recent study identifying the genetic factors controlling flower-size plasticity at different temperatures, the authors also showed that the genetic control of the plasticity differed from the control of flower size itself (Wiszniewski et al., 2022). The specificity of QTL controlling phenotypic plasticity has also been observed in crops, such as tomato (Diouf et al., 2020; Bineau et al., 2021) and maize (Kusmec et al., 2017). If minimising the interaction between the epigenotype and the environment would assure a stable resistance in several environmental conditions, looking for lines particularly plastic for one condition could also be relevant in plant breeding.

While the control of disease resistance traits by transgenerational epimutations has already been demonstrated (Furci et al., 2019; Liégard et al., 2019; Gravot et al., 2024), our study reported for the first time the modulation by environmental conditions of epigenetic factors underlying partial clubroot resistance in *A. thaliana*. We have shown an epigenetic control of the phenotypic variability under all constraint conditions. Although further characterisation and validation of the epigenetic factors detected here as involved in clubroot resistance are still required, our work highlights the potential uses of epigenetic diversity to improve biotic stress resistance in the context of climate change (Gahlaut et al., 2020; Dalakouras and Vlachostergios, 2021). The role of heritable DNA methylation in the control of complex traits was already shown in crops of high agronomical interest such as *Brassica* (Long et al., 2011) or trees like *Populus* (Lu et al., 2020). The search for epimutations associated with clubroot resistance in *Brassica* crops could help to better understand the involvement of epigenetic factors in plant immunity. Taken together, all these results open the prospect of using epigenetic factors in plant breeding.

## Data availability statement

The datasets presented in this study can be found in online repositories. The names of the repository/repositories and accession number(s) can be found below: https://entrepot.recherche.data.gouv.fr/, https://doi.org/10.57745/GSZHJO.

## Author contributions

MP, RL, MLD MJ, JL and CLar carried out the experiments and collected the data. MP, RL, ML, JL and CLar carried out the molecular biology work. MP carried out the genetic analyses and the bioinformatics analyses. CLan help with the monitoring of the experiment. MP, MM-D and MJ wrote the article and MP, MM-D and MJ designed and coordinated the study. All authors contributed to the article and approved the submitted version.
