# Supplementary material for: Environmental conditions modulate the effect of epigenetic factors controlling the response of Arabidopsis thaliana to Plasmodiophora brassicae
Source: Front Plant Sci. 2024 May 29;15:1245545. doi: 10.3389/fpls.2024.1245545 (PMC11171141; doi:10.3389/fpls.2024.1245545)
Supplement: Supplementary file 2 [file Presentation_1.pdf]

Supplementary Figures

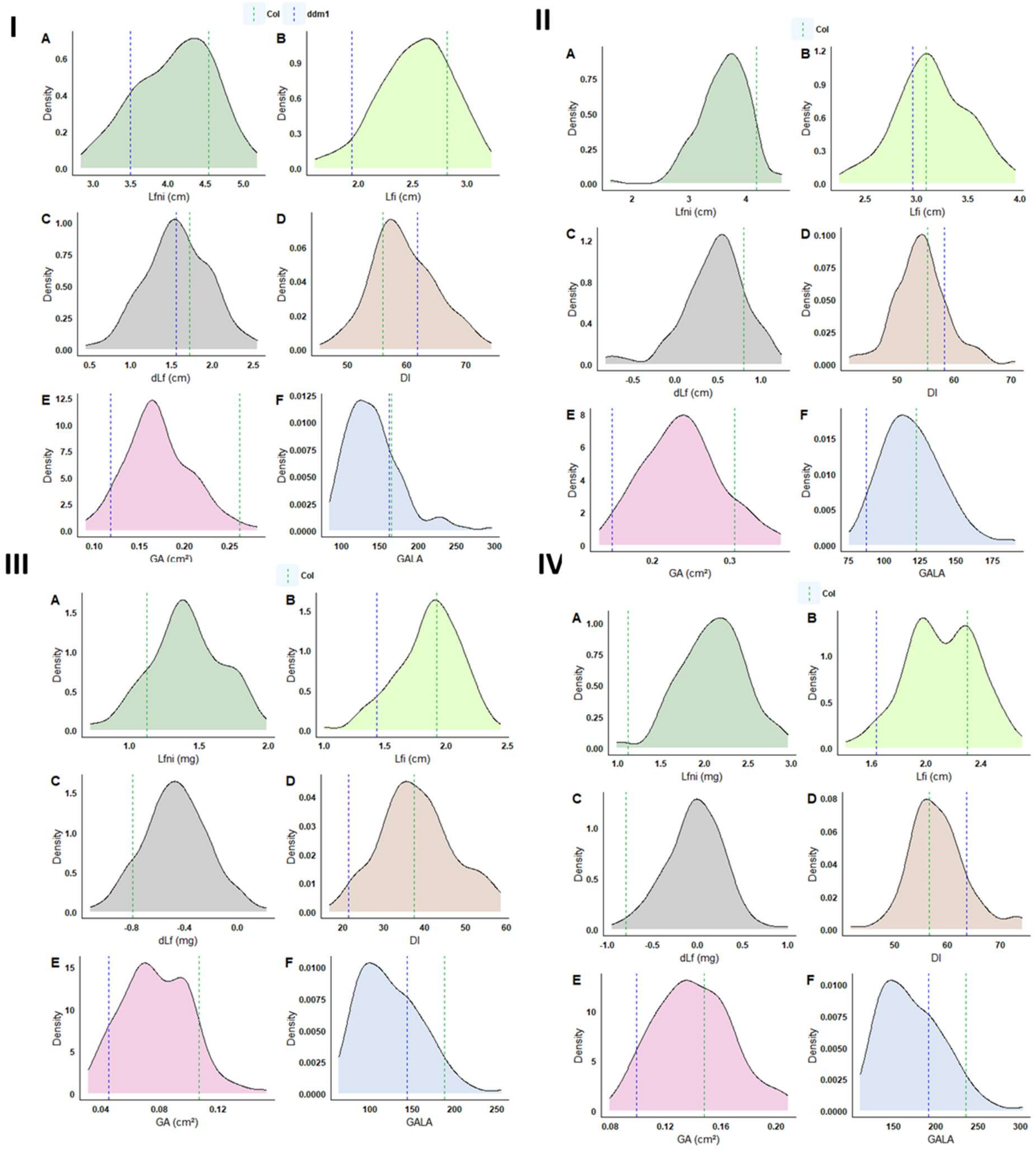

Supp. Figure 1: Distributions of developmental and disease-related traits under I) STANDARD, I) HEAT, III) DROUGHT and IV) FLOODING conditions with A) Lfni, B) Lfi, C) dLf D) DI, E) GA and F) GALA. Green and blue dashed lines correspond to the phenotypical values of the parental lines Col-0 and *ddm1-2*, respectively.

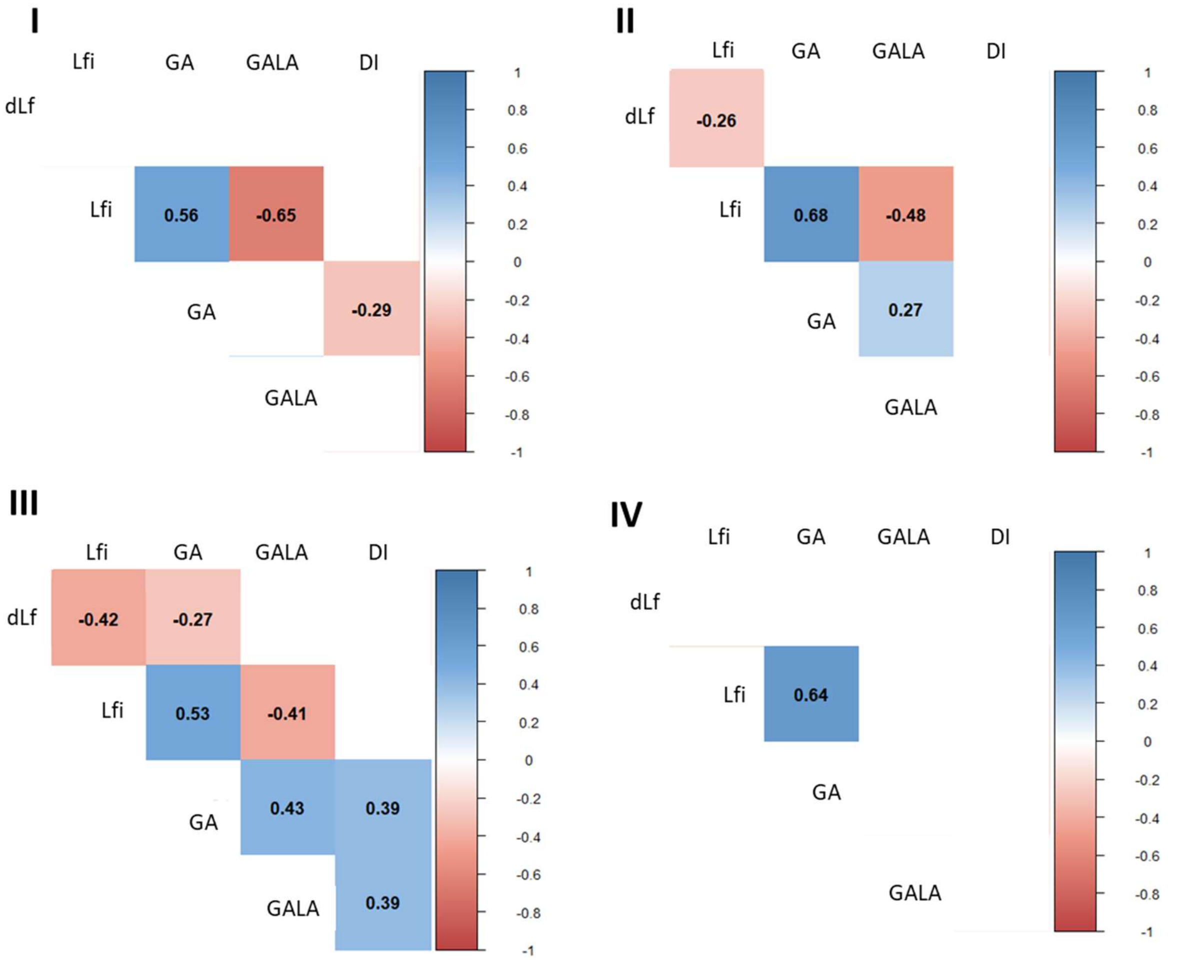

Supp. Figure 2: Correlations between all traits for each condition in I) STANDARD, II) HEAT, III) DROUGHT and IV) FLOOD conditions. Only the significant correlations are presented with *p*-value<0.05.

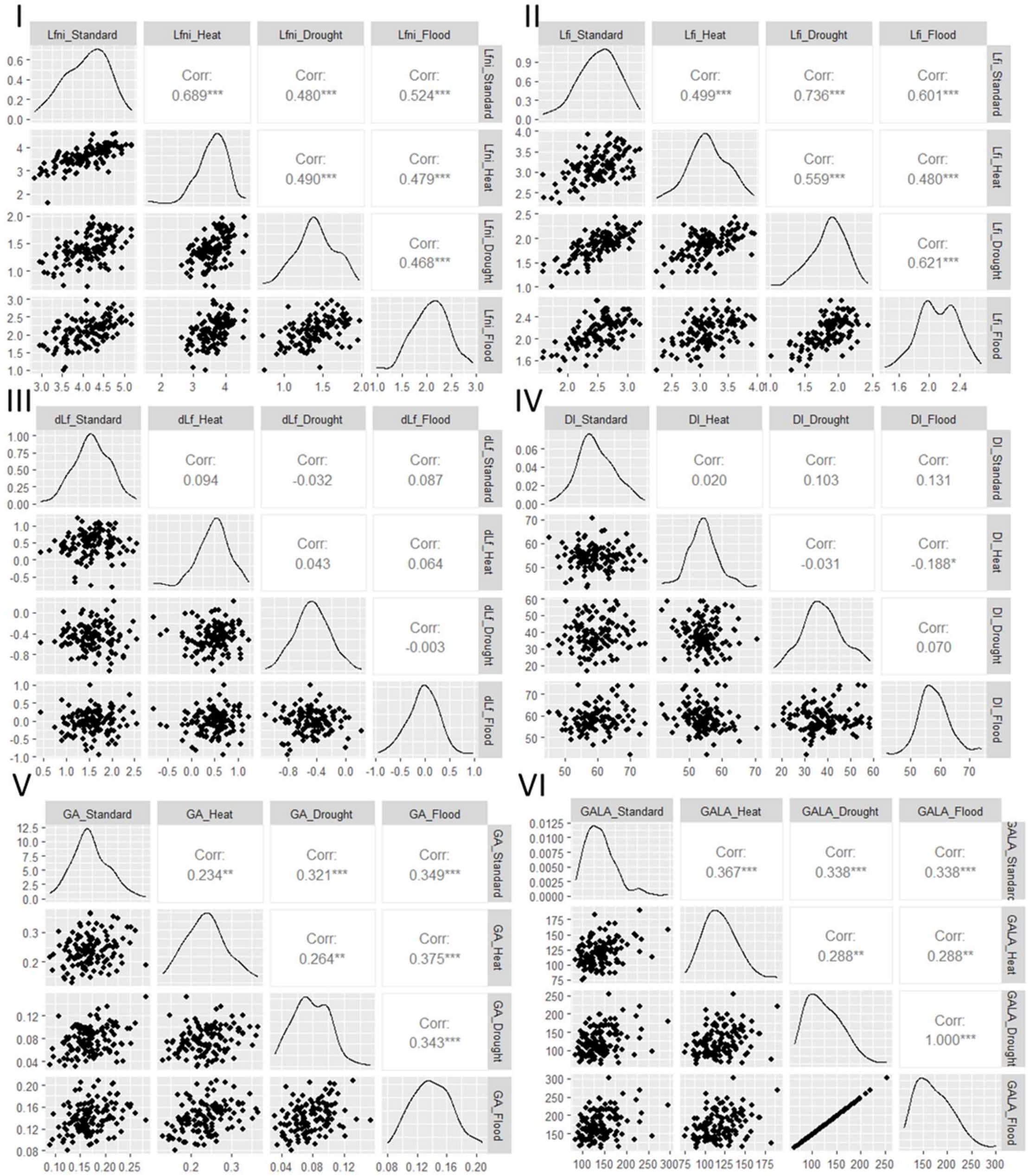

Supp. Figure 3: Correlations between all conditions for each trait with I) Lfni, II) Lfi, III) dLf, IV) DI, V) and VI) GALA.

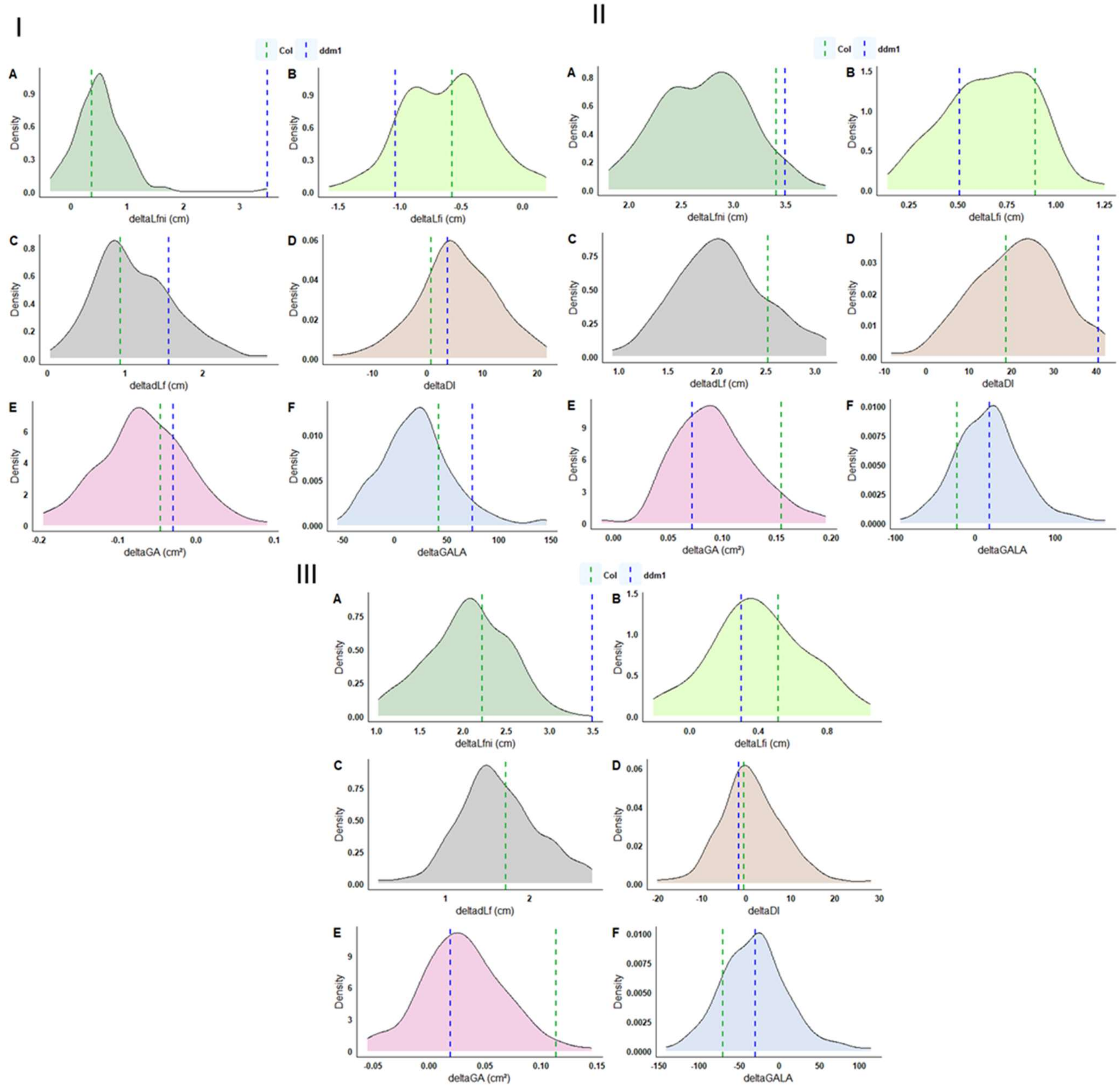

Supp. Figure 4: Distributions of A) deltaLfni, B) deltadLf, C) deltaLfi, D) deltaDI, E) deltaGA and F) deltaGALA for the comparisons between I) STANDARD and HEAT, II) STANDARD and DROUGHT and III) STANDARD and FLOOD. Green and blue dashed lines correspond to the phenotypical values of the parental lines Col-0 and *ddm1-2*, respectively.

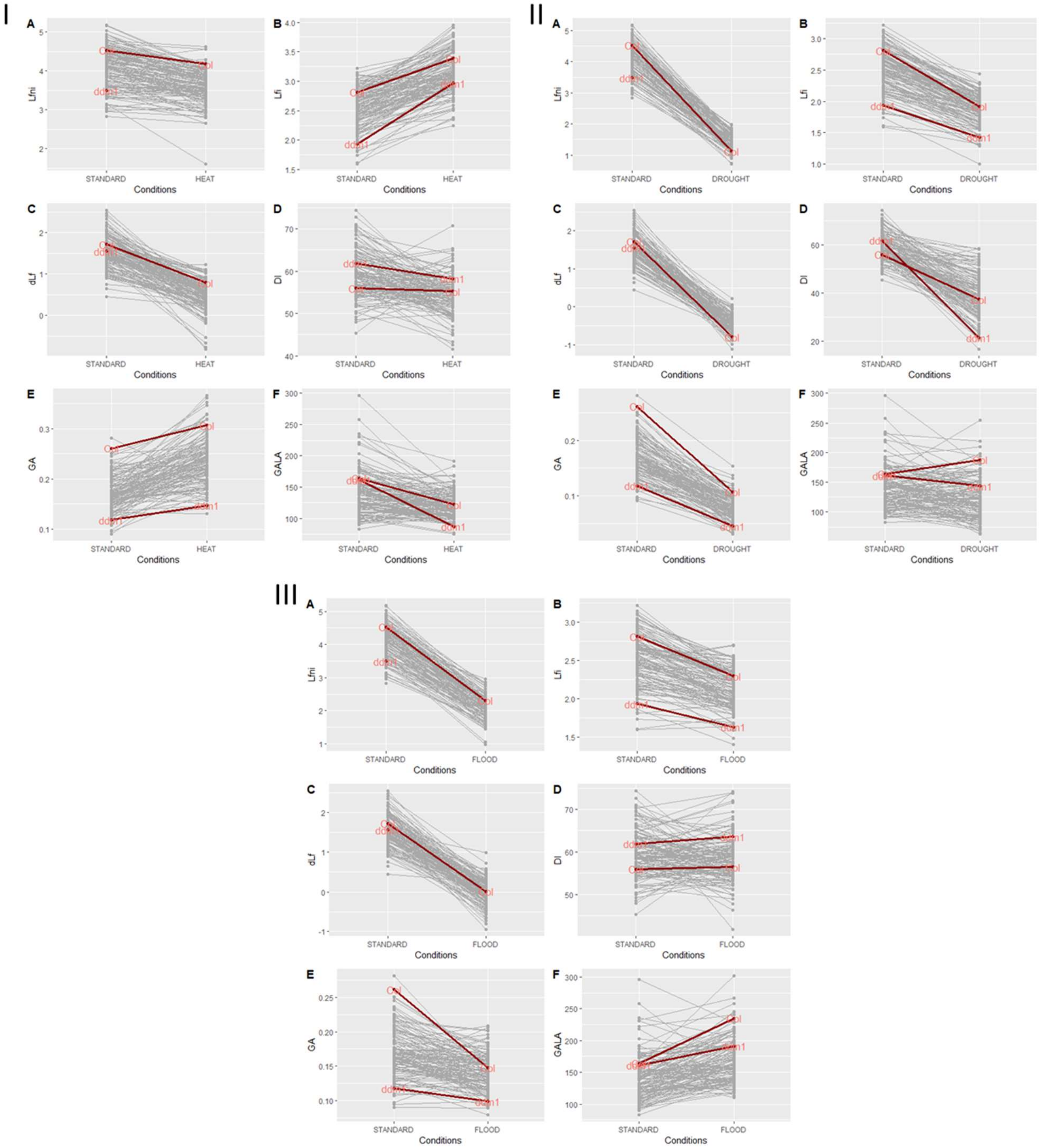

Supp. Figure 5: Reaction Norm plots of epiRIL traits between STANDARD and HEAT (I), STANDARD and DROUGHT (II) and STANDARD and FLOOD (III). In red are given the parental lines Col-0 and *ddm1-2*. A) Lfni, B) Lfi, C) dLf, D) DI, E) and F) GALA variables.

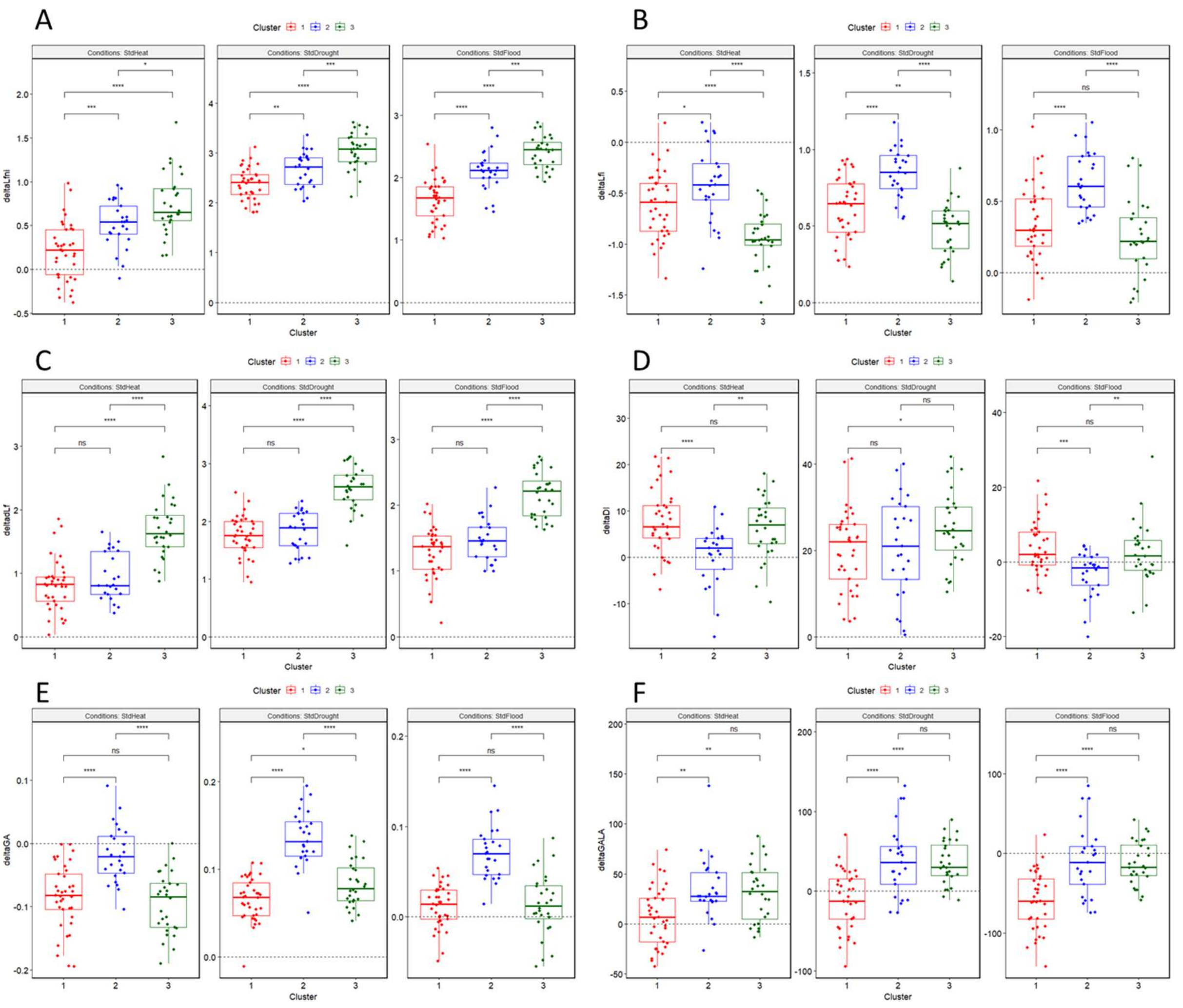

Supp. Figure 6: Boxplots displaying values for 91 epiRIL displaying a cos^2>0.4) with A) deltaLfni, B) deltaLfi, C) deltadLf, D) deltaDI, E) deltaGA and F) deltaGALA for the StdHeat, StdDrought and StdFlood. Cluster 1 is in red, cluster 2 is in blue and cluster 3 is in green.

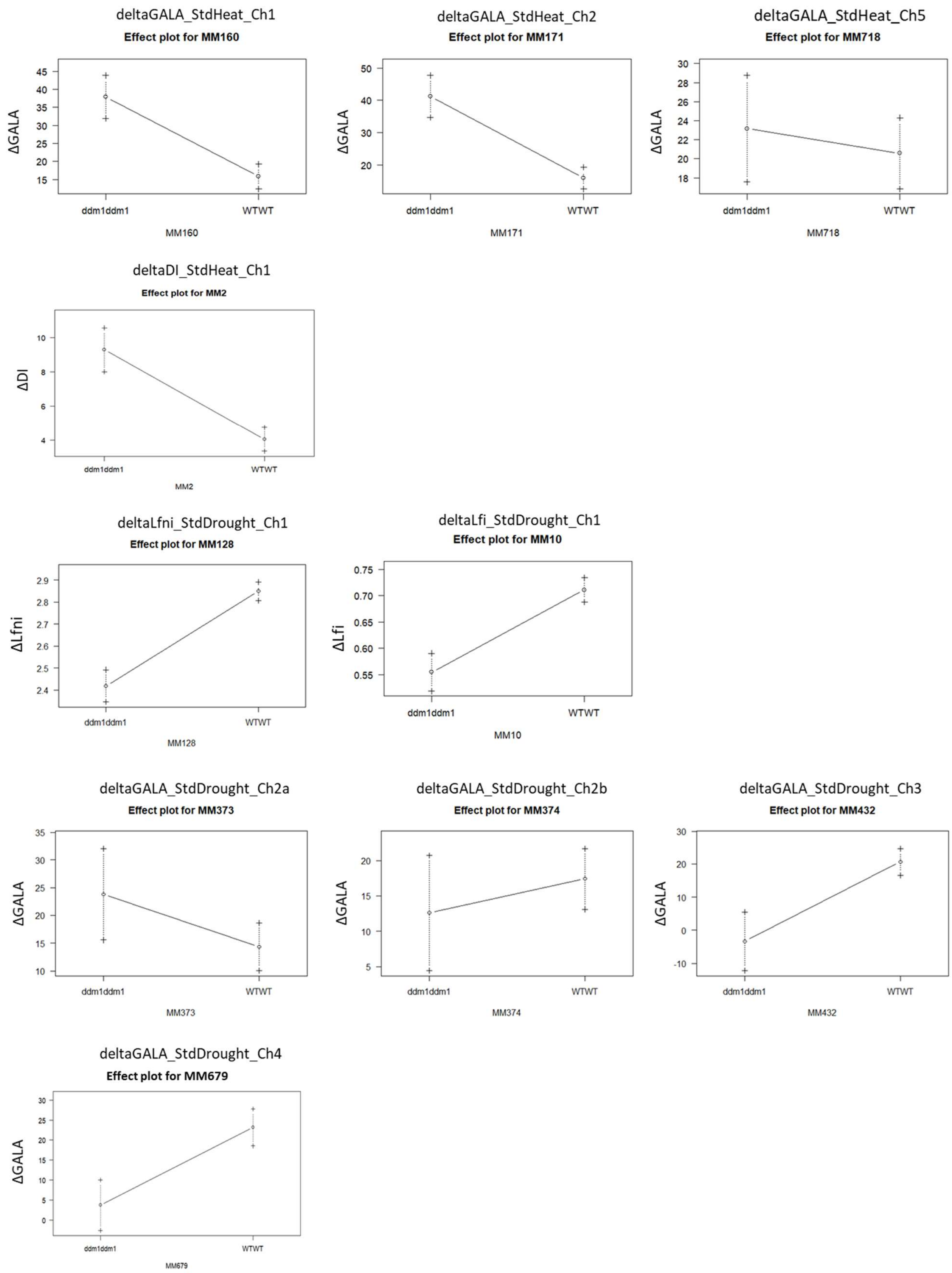

Supp. Figure 7: Effect plots for each QTL^{epi} detected for deltatrait.

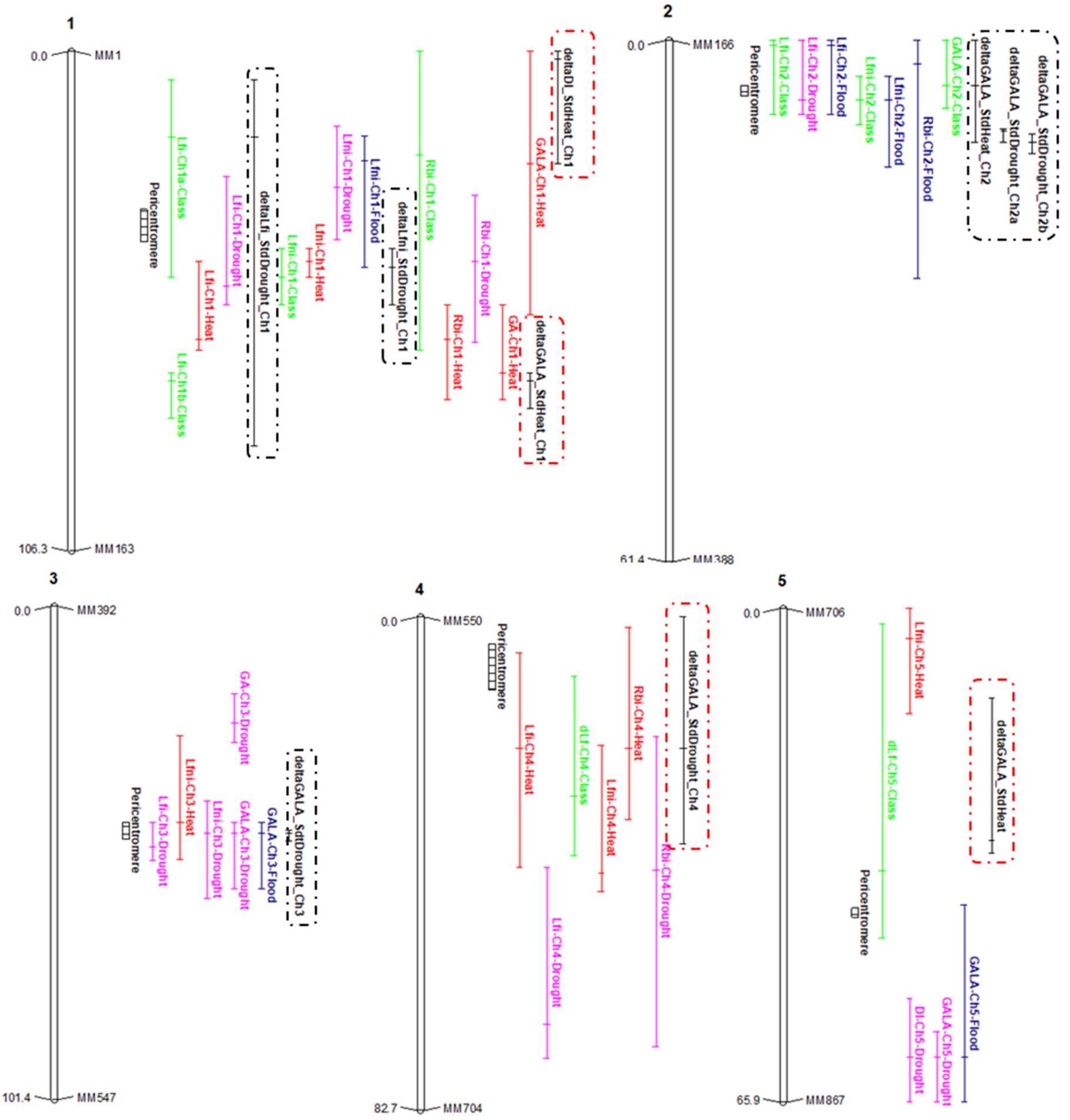

Supp. Figure 8: All QTL^{epi} detected for deltaTrait (in black) overlapping with QTL^{epi} detected for the associated traits (boxed in black) or not (boxed in red).
